# A Hybrid Catheter Localisation Framework in Echocardiography Based on Electromagnetic Tracking and Deep Learning Segmentation

**DOI:** 10.1155/2022/2119070

**Published:** 2022-10-06

**Authors:** Fei Jia, Shu Wang, V. T. Pham

**Affiliations:** ^1^Faculty of Natural, Mathematical and Engineering Sciences, King's College London, London, UK; ^2^Faculty of Life Science and Medicine, King's College London, London, UK; ^3^Saigon University, Hochiminh, Vietnam

## Abstract

Interventional cardiology procedure is an important type of minimally invasive surgery that deals with the catheter-based treatment of cardiovascular diseases, such as coronary artery diseases, strokes, peripheral arterial diseases, and aortic diseases. Ultrasound imaging, also called echocardiography, is a typical imaging tool that monitors catheter puncturing. Localising a medical device accurately during cardiac interventions can help improve the procedure's safety and reliability under ultrasound imaging. However, external device tracking and image-based tracking methods can only provide a partial solution. Thus, we proposed a hybrid framework, with the combination of both methods to localise the catheter tip target in an automatic way. The external device used was an electromagnetic tracking system from North Digital Inc (NDI), and the ultrasound image analysis was based on UNet, a deep learning network for semantic segmentation. From the external method, the tip's location was determined precisely, and the deep learning platform segmented the exact catheter tip automatically. This novel hybrid localisation framework combines the advantages of external electromagnetic (EM) tracking and the deep learning-based image method, which offers a new solution to identify the moving medical device in low-resolution ultrasound images.

## 1. Introduction

A cardiac interventional procedure, also known as an interventional cardiology procedure, is an important type of minimally invasive surgery that deals with catheter-based treatment of cardiovascular diseases, such as coronary artery diseases, strokes, peripheral arterial diseases, and aortic diseases [1]. Generally, it can be classified into the following categories: cardiac catheterization, percutaneous coronary intervention, stents, embolic protection, percutaneous valve repair, balloon valvuloplasty, and atherectomy. A catheter is the medical device used in most cardiac interventions that can be inserted into the body, which functionally allows for drainage, administration of fluids or gases, ablation, and other tasks [2]. There are various types of catheters aiming at different medical applications, for example, the ablation catheter is specifically used for tissue ablation with the generated heat on the electrodes, the pacemaker catheter is to help the heart pump, and a central venous catheter is a conduit to give drugs positioned either in a vein near the heart or inside the atrium. As an example of the procedure, a right-heart catheterization to treat atrial fibrillation requires the catheter to be inserted through the femoral vein and the superior vena cava to the right ventricle. The catheter is placed at the exact site inside the chamber, where the tips emit electrical signals that stimulate abnormal heart rhythm, followed by the transmission of a mild and painless radiofrequency energy to the pathway that destroys the targeted lesion area.

Image guidance during cardiac intervention is a key concept to guarantee patient safety while the direct line of sight is inhibited. X-ray imaging, traditionally, dominates the guidance during cardiovascular interventional procedures, but it provides limited views when the interventions involve the myocardium, pericardium, and cardiac valves. Therefore, cardiac ultrasound (echocardiography) was introduced to navigate these challenges. Compared to cardiac X-ray imaging, echocardiography is especially useful for transcatheter puncture, percutaneous mitral valve procedures, and left atrial appendage closure. Echocardiography fulfils the requirements by providing a real-time imaging solution, with both device and cardiac inner structure demonstration simultaneously [3]. There are three types of echocardiography that can be used during the intervention transthoracic echocardiography (TTE), intracardiac echocardiography (ICE), and transoesophageal echocardiography (TEE/TOE). TTE is widely available and portable. It is a noninvasive imaging procedure [4]. However, it possesses limited ability to visualise the back of the heart and is difficult to use during interventional procedures. ICE has also demonstrated great potential for in vivo medical device monitoring, where a thin probe is inserted inside a patient, but this phased array probe is expensive and can only be used once. Additionally, ICE offers no standard views [5]. As a trade-off between the image quality and imaging cost obtained through echocardiography, TOE imaging is commonly chosen during catheter-based intervention. Prior to imaging, the patient lies in the left lateral decubitus position and swallows the probe following the instruction during probe insertion. Mild to moderate sedation is induced in the patient to ease discomfort and to decrease the gag reflex by providing medications, such as midazolam. This makes the ultrasound probe pass easily into the oesophagus.

Currently, 2-D multiplane imaging is the most widely used mode of TOE, providing 20 standard transoesophageal echocardiographic views that can facilitate and provide consistency in training, reporting, archiving, and quality assurance (as published by the American Society of Echocardiography (ASE) and the Society of Cardiovascular Anaesthesiologists (SCA)) [6]. In clinical practice, before localising and tracking the device from echocardiography, a specific standard view should be determined first. For instance, to view the general four chambers, the probe is positioned at the midoesophagus with a zero-degree rotation. It is then placed at the same position with a 40 degrees rotation. The aortic valve short-axis view can be obtained when the probe goes deeper into the stomach. The right ventricle and left ventricle views can be obtained at the same time from the transgastric apical short-axis view.

To ensure that the catheter tip is accurately localised during a safe interventional procedure when obtaining TOE imaging views, a reliable tracking solution is required. Currently, solutions, in general, can be categorized into two classes: the external tracking system and the image-based method. The external tracking system needs to utilise an extra device to determine the catheter tip location; for example, the Bard Access product that employs the tip confirmation system (TCS) displays different electrocardiogram (ECG) signals, corresponding to different catheter locations [7]. However, surgeons require additional time and knowledge to analyse the external device, and occasionally, these external devices are largely affected by clinical environments. In comparison with external tracking methods, an image-based method is more distinct and easier to apply. Consequently, in recent years, the image-based method has attracted a lot of research attention. Previously, many image-based catheter tracking algorithms were performed on X-ray datasets instead of echocardiography datasets because X-ray images, electrodes, or catheter tips possess distinct characteristic features that can be used for tracking and detection. At the same time, these features were vague in an ultrasound, which led to difficulties in localisation using only the image-based method. The classic image-based methods could only be applied to a small number of images. The methods utilised hand-crafted features. On the contrary, with deep learning, image tasks of greater difficulty can be achieved by end-to-end convolutional neural networks (CNN) [8]. Recently, the use of deep learning has been increasing rapidly in the medical imaging field, including computer-aided diagnosis (CAD), radiomics, and medical image analysis [9].

However, the previous external tracking and image-based methods were two distinct localisation solutions to determine the catheter tip and no combination of these two methods was proposed in previous research studies. Driven by the motivation of assisting cardiologists and sonographers to track the catheter tip during the intervention in a radiation-free and accurate way, this paper proposes a hybrid localisation framework on echocardiography images, with a combination of both EM tracking and deep-learning-based image analysis.

The echocardiography images are collected on a 3D-printed, tissue-mimicking cardiac phantom [10] obtained from several standard TOE views with the Philips IE33 ultrasound machine [11]. Prior to ultrasound imaging, the catheter tip is first localised by the NDI EM tracking systems [12] using a pivot calibration [13] with an error of less than 0.1 mm. Following data collection, all echocardiography images are processed with the Python 3 platform, using the UNet [14] automatic segmentation kernel. This hybrid localisation network can provide a reliable reference for new sonographers and doctors during catheterisation.

## 2. Materials and Methods

### 2.1. Echocardiography Image Collection for Catheter Localisation Model Training

Due to limitations in the availability of the open-source echocardiography dataset and a lack of information to accurately locate the catheter tip, a complete echocardiography image dataset needs to be developed first. To obtain the catheter tip information, at the same time, instead of using real patient data, the cardiac interventional procedure was simulated on a 3-D printed Lay-Fomm 40 phantom, which can fully resemble an adult patient heart. During imaging, the Philips S5-1 broadband sector array probe was placed on top of the cardiac phantom, while the phantom was fixed at the bottom of a plastic water tank. Subsequently, several TOE standard views were acquired by probe manipulation, such as the upper aortic valve view (commonly chosen in a real patient case). During the simulation, the Philips IE33 ultrasound machine was set to full volume mode with each image acquisition lasting for five seconds. All the DICOM images were then exported to blank CDs and analysed with ITK-SNAP [15].

**Figure 1:**
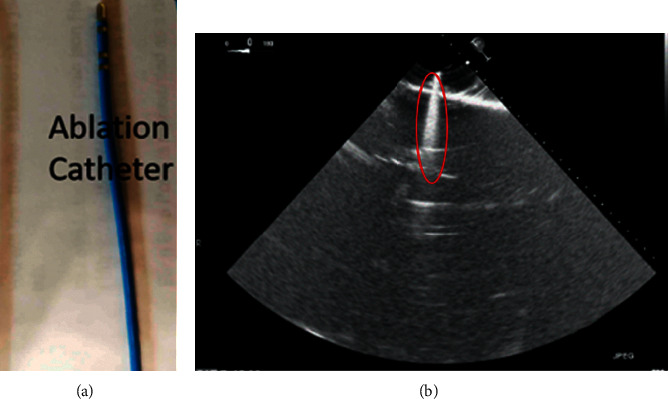
(a) Shelley medical ablation catheter. (b) Corresponding 2-D ultrasound image of (a).

**Figure 2:**
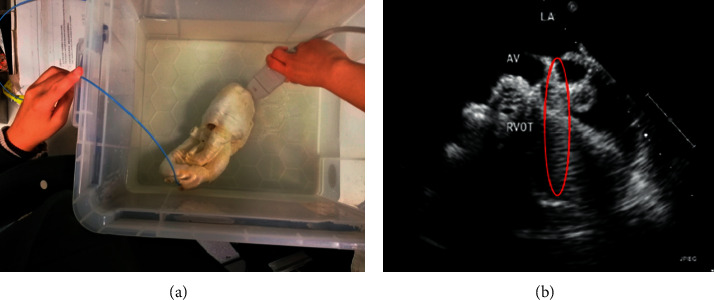
Catheter tip movement. (a) 2-D echocardiography image acquisition on 3-D printed cardiac phantom. (b) Corresponding ultrasound results of (a) under aortic valve short-axis view.

The corresponding ultrasound imaging results of the Shelley medical ablation catheter movements can be observed in Figure 1, in which the vertical line (circled area) indicates the catheter shape. While the horizontal line is the artefact, which will not be labelled in the following works. In Figure 2, we can observe from the echo image that the background contained both the ablation catheter and the cardiac structures. In the aortic valve, the visualisation of the catheter is not only affected by the valve structure but also affected by strong reverberations from the water tank. The low-image resolution also increases the difficulty to localise the catheter tip accurately.

### 2.2. Catheter Tip Determination via the External EM Device and Pivot Calibration

Before applying the deep learning network in image-based methods for automatic catheter segmentation, it was necessary to determine the exact location of the tracked catheter tip in the trained ultrasound dataset to provide the groundtruth. As illustrated in Figures 1 and 2, it is usually difficult to identify the catheter tip in the image by visual inspection alone. Therefore, mapping the physical location of the catheter tip to where it appears in the ultrasound imaging provided an alternative approach to obtain reliable groundtruth.

**Figure 3:**
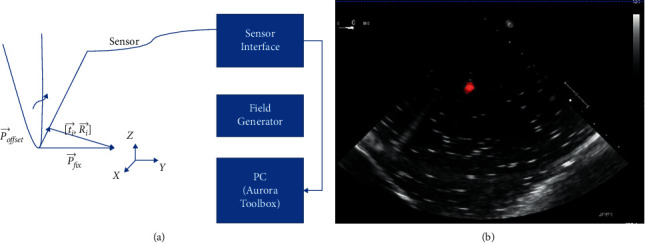
(a) Illustration of pivot calibration using the NDI Aurora EM tracking system. (b) Mapped location of catheter tip in 2D echocardiography.

In this section, the NDI Aurora EM tracking system [12] depicted in Figure 3(a) was used to arrive at the catheter tip's location physically because of its nonradiation and real-time 3-D tracking ability. This system consists of an EM field generator and a sensor interface unit to connect the sensor and the system control unit (SCU) connected to the computer. To simplify the setup, a 6-degree of freedom (DOF) catheter-type EM sensor from NDI was used to represent the catheter, as it could generate a mapped point on a 2-D ultrasound image as indicated in Figure 3(b). The Shelley medical ablation catheter could not be directly connected to the EM tracking device. It was tied to the sensor so that the tracked tip location could be shared. The physical location of the tip was calculated through a pivot calibration experiment. The use of external EM tracking is to determine the length of the catheter tip before training the lateral network, without EM tracking, the catheter tip cannot be defined during ground-truth labelling.

During tracking, all the data was recorded by the EM tracking device, saved as a text file, and then processed further in MATLAB. Initially, the EM tracking device recorded the sensor's 3-D location with the help of the tracking system coordinates. Through pivot calibration, the sensor was manually moved around a fixed pivot point near the EM field generator. The corresponding location matrix in the tracking system coordinates was transformed through

(1) $$\begin{array}{c} \left(x,y,z\right)=\overset{⟶}{t_{i}},\left(q_{0},q_{x},q_{y}\;\right)=\overset{⟶}{R_{i}}, \end{array}$$

(2) $$\begin{array}{c} \overset{⟶}{R_{i}}\cdot {\overset{⟶}{P}}_{\text{offset}}+\overset{⟶}{t_{i}}={\overset{⟶}{P}}_{\text{fix}}, \end{array}$$

(3) $$\begin{array}{c} \overset{⟶}{R_{i}}\cdot {\overset{⟶}{P}}_{\text{offset}}-{\overset{⟶}{P}}_{\text{fix}}\cdot \overset{⟶}{I}=-\overset{⟶}{t_{i}}, \end{array}$$

(4) $$\begin{array}{c} \left[\begin{array}{cc} \overset{⟶}{R_{1}} & -\overset{⟶}{I}\\ \overset{⟶}{R_{2}} & -\overset{⟶}{I}\\ \overset{⟶}{R_{N}} & -\overset{⟶}{I} \end{array}\right]\cdot \left[\begin{array}{cc} {\overset{⟶}{P}}_{\text{offset}} & {\overset{⟶}{P}}_{\text{fix}} \end{array}\right]=\left[\begin{array}{c} -\overset{⟶}{t_{1}}\\ -\overset{⟶}{t_{2}}\\ -\overset{⟶}{t_{N}} \end{array}\right], \end{array}$$

(5) $$\begin{array}{c} \left[\begin{array}{cc} {\overset{⟶}{P}}_{\text{offset}} & {\overset{⟶}{P}}_{\text{fix}} \end{array}\right]=p\text{suedo\_inverse}\left[\begin{array}{cc} \overset{⟶}{R_{1}} & -\overset{⟶}{I}\\ \overset{⟶}{R_{2}} & -\overset{⟶}{I}\\ \overset{⟶}{R_{N}} & -\overset{⟶}{I} \end{array}\right]*\left[\begin{array}{c} -\overset{⟶}{t_{1}}\\ -\overset{⟶}{t_{2}}\\ -\overset{⟶}{t_{N}} \end{array}\right]. \end{array}$$

### 2.3. Automatic Catheter Segmentation in 2-D Echocardiography through Deep Learning

After determining the location of the ablation catheter tip physically, the state-of-the-art UNet was utilised to train the deep learning-based automatic segmentation platform on the collected phantom echocardiography from different standard TOE views [16, 17]. To make the trained model more robust, another 19 real patient TOE folders were mixed and tested at the same time.

**Figure 4:**
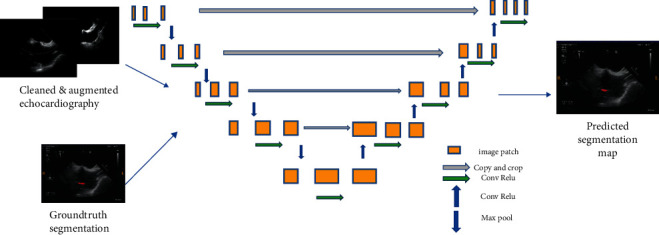
Diagram of the deep learning model trained on 2-D echocardiography dataset.

Most of the image-based localisations mentioned in the literature were performed either through tracking with an inaccurate bounding box or by locating landmarks from registration. Both of these methods lacked the target shape information. However, semantic segmentation could solve the difficulty associated with an accurate localisation. Currently, the state-of-the-art semantic segmentation model being employed is UNet [18–23], which is depicted in Figure 4 [14]. This model can be used on smaller datasets, such as medical images for faster training, while the deep learning models have to be trained on larger datasets with more variations. Unlike a model based on CNN, which can only predict probability distribution, UNet is built with a fully convolutional network (FCN) [17] kernel. It can, thus, directly provide a full and accurate output segmentation map on the image.

**Figure 5:**
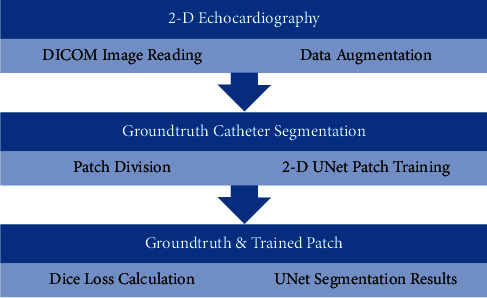
Flow chart of echocardiography UNet model training procedure.

The segmentation model was built on 2-D TOE images, collected from the Lay-Fomm 40 cardiac phantom, fabricated prior to obtaining both standard and nonstandard views, with the ablation catheter moving from random places in the image. The image dataset contained 20 image volumes with 75 slices for each volume. The example provided in Figure 4 is a bicaval view with the catheter segmentation in the right atrium, and all of the ground-truth labels were obtained from the doctors' manual segmentation (under the reference of both EM tracking results and visual inspections). The segmentation algorithm is written in Python 3. As illustrated in Figure 5, the model training procedure is given, which is consisted of the following main steps:

(1). DICOM image reading and data cleaning
(2). Data augmentation to enlarge the training dataset
(3). Obtain a random positive and negative patch
(4). Train and validate the patch based on 2-D UNet
(5). Generate the Dice loss plot for parameter tuning
(6). Test and generate the predicted segmentation

The parameters used in the UNet segmentation model [24–32] were as follows:

(1). First ten volumes as the training dataset, second set of ten volumes as the validation dataset, and testing on 14 random 2-D echocardiography volumes
(2). Dropout at the last layer with the rate of 0.5
(3). Augmenting the data offline to ten times as before
(4). Use early stopping with the patience of 6000 iterations
(5). Positive and negative rate of 0.95 (when the image slice contained the segmentation target, we regarded it as a positive slice)
(6). Patch size of 1,448,448
(7). Batch size of four
(8). Total number of iterations (batch size) was 30000
(9). Iterative UNet depth of five
(10). Loss function: Dice loss and Laplace smoothing for preventing overfitting
(11). Activation function as ReLU

To better describe the performance by automatic segmentation when compared to ground-truth labelling, we introduced a Dice loss to evaluate how accurate the prediction would be (calculated through equation [37]).

(6) $$\begin{array}{c} \text{Dice loss}=1-\frac{2\left|X\cap^{}Y\right|}{\left|X\right|+\left|Y\right|}=\frac{2TP}{2TP+FP+FN}=1-\text{Dice accuracy}. \end{array}$$

In the above equation, *X* is the predicted segmentation, *Y* is the true segmentation, *TP* stands for true positive, and *FP* and *FN* are false positive and false negative rates, respectively. The Dice accuracy results on the testing dataset will be demonstrated in the Results and Discussion section.

## 3. Results and Discussion

**Figure 6:**
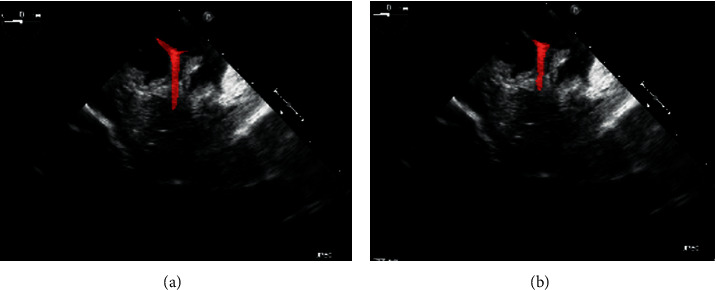
(a) Ground-truth segmentation of Shelley medical ablation catheter. (b) Deep learning platform predicted catheter segmentation on simulated 2-D echocardiography.

**Figure 7:**
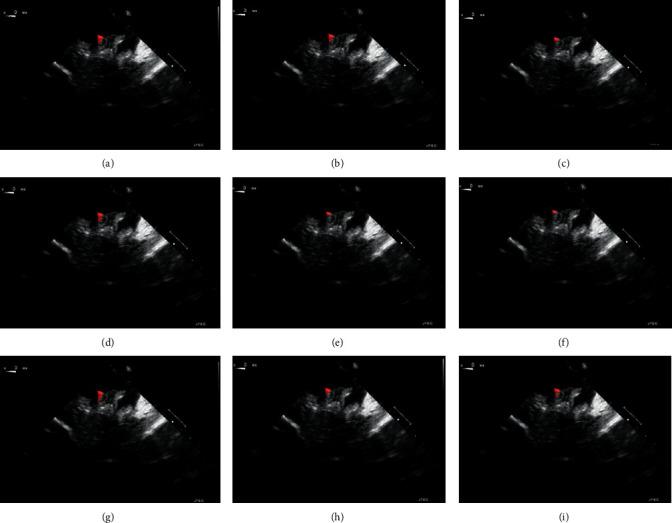
Catheter tip segmentation results by deep learning platform in 2-D echocardiography sequences: (a) 0.1 s, (b) 0.2 s, (c) 0.3 s, (d) 0.4 s, (e) 0.5 s, (f) 0.6 s, (g) 0.7 s, (h) 0.8 s, and (i) 1 s.

The physical localisation result of the targeted ablation catheter was 4.7362 ± 0.3523 mm, and the corresponding catheter's ground-truth segmentation is indicated in Figure 6(a).  Figure 6(b) indicates the corresponding prediction results of Figure 6(a), trained by the deep learning platform. The original 2-D echocardiography of Figure 6 is the simulated result collected on the 3-D printed cardiac phantom. With the proposed hybrid framework, the accuracy of the catheter tip's groundtruth location can be guaranteed at 0.1 mm. When compared to the traditional groundtruth by considering a doctor's visual inspection alone, this new groundtruth is more reliable. During the catheter movement, the final trained model could still identify the dynamic target as indicated in Figure 7. Within one second, no catheter tip was missing in every single frame, but at the same time, as the current deep learning network has a limited ability in recognizing moving target shown in Figure 7, some predicted segmentations are incomplete compared to the groundtruth. The corresponding Dice accuracy results are as follows:

(7) $$\begin{array}{c} 0.8138811737712837\;\left(\max\right),0.6771465049473349,0.8079201691686618,\\ 0.7211444100551068,\;\;0.7322477650063857,\;0.7824308664136881,\\ 0.8112021032810474,\;0.6286978766145106\;\left(\min\right),\;\;0.7643511925952297. \end{array}$$

**Figure 8:**
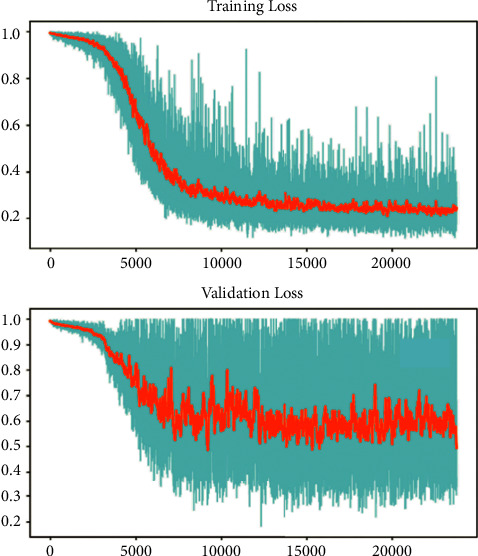
Training curve of the deep learning model's training and validation loss.

The training and testing of the Dice loss plots, indicated in Figure 8, are consistent with the aforementioned accuracy results. The Dice loss on the training dataset was rather low. However, on the validation dataset, the Dice loss rose significantly (which indicated that the trained model was overfitting to a certain extent). Upon comparing the UNet model accuracy with other high-image quality datasets, the lower prediction accuracy could be explained by the target being too sparse and ambiguous to identify. Therefore, the accuracy obtained cannot compete with the performance on CT or MRI volumes. Except for the difficulty faced (due to the target being sparse or ambiguous), another challenge attributed to the limited variation of TOE images obtained caused the overfitting of the model, which was unavoidable.

**Figure 9:**
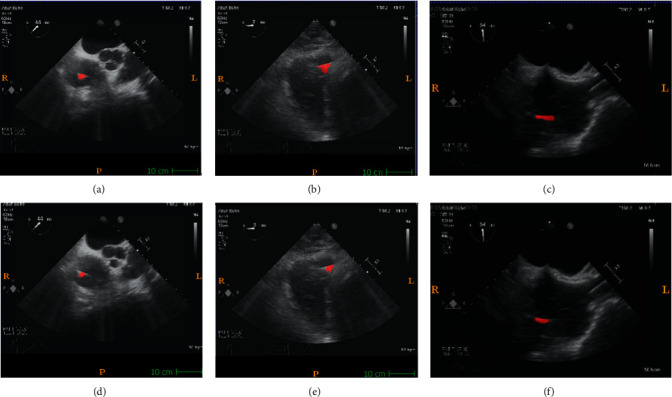
Validation of the model on real patient echocardiography: (a–c) groundtruth segmentation from ME-RV inflow, TG Basal SAX, and bicaval view; (d–f) deep learning predicted segmentation of (a–c).

To validate the trained model's ability for generalisation, the model was also tested with real patient data obtained from several standard TOE views, such as the midoesophagus right ventricle (ME-RV) inflow view and the transgastric basal short-axis (TG basal SAX) view. From Figure 9, the predicted results proved the generalisation ability of the proposed model with the correct catheter location and shape. As the target was too blurred, the shape may have varied from the groundtruth to a certain level.

## 4. Conclusions

EM tracking device is easily get affected by the clinical environment, and it cannot provide visual information about the medical device; while the target in medical images cannot provide numerical results. This new hybrid localisation framework combines the advantages of external EM tracking and deep-learning-based image methods, and successfully builds up the connection between the physical coordinate and the image coordinates, which offers a new solution for obtaining a more reliable groundtruth to train the automatic deep learning model. At the same time, 3-D printed phantoms also provide a new direction for collecting the original dataset to train the deep learning models based on our requirements.

Based on the simulated dataset and EM tracking tip determinations, the reliability of deep-learning-based models can be guaranteed. However, the model's accuracy and stability need to be improved in the future. During future improvement, the groundtruth labels have to be derived from the EM sensor, while all the possible standard views need to be classified too. Due to the dataset limitations, all the networks built thus far faced the overfitting problem, so an adequate fully automatic solution for cardiac intervention has not yet been achieved.

The future plan will be to try and optimise this platform from the following perspectives: TOE standard view classification and learning and EM tracking groundtruth labelling. To improve manual inspection, an automatic EM tracking mapping system will be developed so that the automatic labelling of the groundtruth can replace the current semiautomatic method. Increased real patient data will be involved and analysed (including the condition of the patients) with a variety of different machines, including catheters. Meanwhile, the deep learning model will also be optimised into a more stable and accurate one, which can adapt to accommodate a larger patient dataset.

## Data Availability

The dataset can be accessed upon request.

## References

[1] Wu X. (2014). Fast Catheter segmentation and tracking based on X-ray fluoroscopic and echocardiographic modalities for catheter-based cardiac minimally invasive interventions. *PhD Thesis*.

[2] Diggery R. (2012). *Catheters: Types, Applications and Potential Complications Medical Devices and Equipment*.

[3] Wunderlich N., Franke J., Wilson N., Sievert H. (2009). 3D echo guidance for structural heart interventions. *Interventional Cardiology Review*.

[4] Silvestry F. E., Kerber R. E., Brook M. M. (2009). ASE recommendations for clinical practice: echocardiography-guided interventions. *Journal of the American Society of Echocardiography*.

[5] Jongbloed M. R. M., Schalij M. J., Zeppenfeld K., Oemrawsingh P. V., Van Der Wall E. E., Bax J. J. (2005). Clinical applications of intracardiac echocardiography in interventional procedures. *Heart*.

[6] Shanewise J. S., Cheung A. T., Aronson S. (1999). ASE/SCA guidelines for performing a comprehensive intraoperative multiplane transesophageal echocardiography examination: recommendations of the American society of echocardiography Council for intraoperative echocardiography and the society of cardiovascular anesthesiologists task force for certification in perioperative transesophageal echocardiography. *Anesthesia & Analgesia*.

[7] Johnston A. J., Holder A., Bishop S. M., See T. C., Streater C. T (2014). Evaluation of the Sherlock 3CG Tip Confirmation System on peripherally inserted central catheter malposition ratesfirmation System on peripherally inserted central catheter malposition rates. *Anaesthesia*.

[8] Zhou B., Khosla A., Lapedriza A., Oliva A., Torralba A. Learning deep features for discriminative localization.

[9] Suzuki K. (2017). Overview of deep learning in medical imaging. *Radiol Phys Technol*.

[10] Ahangar P., Akoury P., Ramirez Garcia E. (2018). Nanoporous 3D-printed Scaffolds for Local Doxorubicin Delivery in Bone Metastases Secondary to Prostate Cancer. *Materials*.

[11] Philips iE33 (2020). Philips iE33 ultrasound system - avante health solutions. https://avantehs.com/p/philips-ie33-ultrasound-system/12681.

[12] Nafis C., Jensen V., Beauregard L., Anderson P. (2006). Method for estimating dynamic EM tracking accuracy of surgical navigation tools Proc. SPIE 6141. *Medical Imaging 2006: Visualization, Image-Guided Procedures, and Display*.

[13] Yaniv Z. (2015). Which pivot calibration?. *SPIE Proceedings*.

[14] Ronneberger O., Fischer P., Brox T. (2015). U-net: convolutional networks for biomedical image segmentation medical image Computing and computer-assisted intervention – MICCAI 2015. *Lecture Notes in Computer Science*.

[15] Yushkevich P. A., Piven J., Hazlett H. C. (2006 Jul 1). User-guided 3D active contour segmentation of anatomical structures: significantly improved efficiency and reliability. *NeuroImage*.

[16] Virtual T. E. E. (2020). Standard views, cardiac, transesophageal echocardiography, 3D heart model, education. http://pie.med.utoronto.ca/TEE/TEE_content/TEE_standardViews_intro.html.

[17] Shelhamer E., Long J., Darrell T. (2017). Fully convolutional networks for semantic segmentation. *IEEE Transactions on Pattern Analysis and Machine Intelligence*.

[18] Yin X.-X., Sun Le, Fu Y., Lu R., Zhang Y. (2022). U-Net-Based medical image segmentation. *Journal of Healthcare Engineering*.

[19] Li L.-H., Hang J.-C., Gao Y., Mu C.-Y. (2017). Using an integrated group decision method based on SVM, TFN-RS-AHP, and TOPSIS-CD for cloud service supplier selection. *Mathematical Problems in Engineering*.

[20] Li L. H., Hang J. C., Sun H. X., Wang L. (2017). A conjunctive multiple-criteria decision-making approach for cloud service supplier selection of manufacturing enterprise. *Advances in Mechanical Engineering*.

[21] Liu L., Meng L., Zheng W., Peng Y., Wang X. (2022). A Novel High-Capacity Information Hiding Scheme Based on Improved U-Net. *Security and Communication Networks*.

[22] Lin Y. L., Huang A., Yang C. Y., Chang W. Y. (2022). Measurement of body surface area for psoriasis using U-net models. *Computational and Mathematical Methods in Medicine*.

[23] Li L., Mao C. (2020). Big data supported PSS evaluation decision in service-oriented manufacturing. *IEEE Access*.

[24] Li L., Mao C., Sun H., Yuan Y., Lei B. (2020). Digital twin driven green performance evaluation methodology of intelligent manufacturing: hybrid model based on fuzzy rough-sets AHP, multistage weight synthesis, and PROMETHEE II. *Complexity*.

[25] Liu Z., Su B., Lv F. (2022). Intelligent Identification Method of Crop Species Using Improved U-Net Network in UAV Remote Sensing Image. *Scientific Programming*.

[26] Kwon H., Jeong J. (2022). A.U.-N.: Generating adversarial example based on medical image and targeting U-net model. *Journal of Sensors*.

[27] Dou C., Li K., Wang L. (2022). Computed tomography image segmentation of the proximal colon by U-net for the clinical study of somatostatin combined with intestinal obstruction catheter. *Computational and Mathematical Methods in Medicine*.

[28] Li L., Qu T., Liu Y. (2020). Sustainability assessment of intelligent manufacturing supported by digital twin. *IEEE Access*.

[29] Sun R., Chang R., Yu T., Wang D. (2022). U-net modelling-based imaging MAP score for Tl stage nephrectomy: an exploratory study. *Journal of Healthcare Engineering*.

[30] Li L., Lei B., Mao C. (2022). Digital twin in smart manufacturing. *Journal of Industrial Information Integration*.

[31] Wang Y., Kong J., Zhang H. . U.-N. (2022). A Smart Application with Multidimensional Attention Network for Remote Sensing Images. *Scientific Programming*.

[32] Fei J., Shu W. (2022). A hybrid catheter localisation framework in echocardiography based on electromagnetic tracking and deep learning segmentation. https://www.medrxiv.org/content/10.1101/2020.12.22.20248705v1.full.pdf.

